# Potential of algal-based products for the management of potato brown rot disease

**DOI:** 10.1186/s40529-023-00402-y

**Published:** 2023-10-16

**Authors:** Seham M. Hamed, Marwa Kamal, Nevein A. S. Messiha

**Affiliations:** 1 Soil Microbiology Department, Soils, Water and Environment Research Institute, Agricultural Research Centre (ARC), P.O. 175, Giza, El‒Orman, Egypt; 2 Botany and Microbiology Department, Faculty of Science, Beni-Suef University, Beni-Suef, 62521 Egypt; 3 Bacterial Diseases Research Department, Plant Pathology Research Institute, Agricultural Research Centre (ARC), Giza, Egypt

**Keywords:** *Ralstonia solanacearum*, Biostimulants, Seaweeds, *Spirulina platensis*, Antioxidants, Organic farming, Microbial biodiversity

## Abstract

**Background:**

*Ralstonia solanacearum* causes potato brown rot disease, resulting in lower crop’s production and quality. A sustainable and eco-friendly method for controlling the disease is required. Algae’s bioactive chemicals have shown the potential to enhance plant defenses. For the first time, the efficacy of foliar application of *Acanthophora spicifera* and *Spirulina platensis* seaweed extracts, along with the utilization of dried algal biomasses (DABs) of *Turbinaria ornata* and a mixture of *Caulerpa racemosa* and *Cystoseira myrica* (1:1)on potato yield and brown rot suppression were investigated under field conditions. Field experiments were conducted in three locations: Location 1 (Kafr Shukr district, Kaliobeya governorate), Location 2 (Moneira district, Kaliobeya governorate), and Location 3 (Talia district, Minufyia governorate). Locations 1 and 2 were naturally infested with the pathogen, while location 3 was not. The study evaluated potato yield, plant nutritive status and antioxidants, soil available nitrogen-phosphorus-potassium (N-P-K), and organic matter percentage. Additionally, the shift in soil microbial diversity related to *R. solanacearum* suppression was examined for the most effective treatment.

**Results:**

The results revealed that seaweed extracts significantly increased potato yield at all locations, which correlated with higher phosphorus absorption, while *T. ornate* DAB increased potato yield only at location 2, accompanied by noticeable increases in soil nitrogen and plant phosphorus. The mixed DABs of *C. racemosa* and *C. myrica* demonstrated greater disease suppression than foliar applications. The disease-suppressive effect of the mixed DABs was accompanied by significant increases in flavonoids and total antioxidant capacity (TAC). Moreover, the application of mixed DABs increased soil bacterial biodiversity, with a higher abundance of oligotrophic marine bacterial species such as *Sphingopyxis alaskensis* and growth-promoting species like *Glutamicibacter arilaitensis*, *Promicromonospora* sp., and *Paenarthrobacter nitroguajacolicus* in all three locations compared to the untreated control. *Klebsiella* sp., *Pseudomonas putida*, and *P. brassicacearum* abundances were increased by the mixed DABs in Location 1. These species were less abundant in locations 2 and 3, where *Streptomyces* sp., *Bacillus* sp., and *Sphingobium vermicomposti* were prevalent.

**Conclusions:**

The results demonstrated that the used seaweed extracts improved potato yield and phosphorous absorption, while the mixed DABs potentially contributed in disease suppression and improved soil microbial diversity.

**Supplementary Information:**

The online version contains supplementary material available at 10.1186/s40529-023-00402-y.

## Background

The potato (*Solanum tuberosum* L.) holds a paramount position as a vegetable crop in human diets. Its introduction to Egypt in the early 19th century, lead to its widespread cultivation throughout the country. Owing to its high nutritional value, the potato quickly became a staple dish, commonly served in cooked, crispy, or chip form. Egypt is a significant potato exporter in Africa and among the top 20 potato growers in the world (Rabia et al. 2021). The average potato yield in Egypt has reached approximately 5.2 million tons, cultivated across approximately171 thousand hectares (FAO 2015). The primary potato production area in Egypt, constituting 65% of the total, is located in the Nile Delta and the Valley governorates (old districts) due to the suitable climate and soil fertility (FAOSTAT 2018; AbdEl-Hady and Abdelaty, 2019).

In fact, the potato crop in old districts in Egypt is frequently prone to brown rot disease (bacterial wilt) caused by *Ralstonia solanacearum* race 3, biovar 2, Phylotype II, sequevar 1 (Prior and Fegan 2005; Messiha 2006; Messiha et al. 2021). Nonetheless, the European Union (EU) has imposed strict importation restrictions on Egypt (European Communities, 2005). These restrictions require the growing of potatoes designated for the EU inside approved Pest-Free Areas (PFAs), which are predominantly located in desert regions, and exclude the Nile Delta and Valley region from export activities. This devastating soil-borne pathogen is reported in tropical, subtropical, and temperate regions of the world, causing pronounced losses in different crop hosts (Farag et al. 2017; Messiha et al. 2019). Previous reports indicated that the chemical control methods such as soil fumigants, copper-containing pesticides, and antibiotics had doubtful effects (Farag et al. 1986), besides their hazardous negative ecological impacts (Hartman and Elphinstone 1994).

Numerous attempts have been undertaken to directly control *R. solanacearum* by utilizing antagonistic microbes or their extracts (Elhalag et al. 2016; Hamed and Messiha 2018). Indirect approaches involve the application of mineral and organic fertilisers (Messiha et al. 2021) or the adoption of biological soil disinfestation (BSD) which creates anaerobic soil conditions to impede phytopathogen proliferation (Blok et al. 2000; Messiha et al. 2007). Furthermore, previous research has explored the use of natural antioxidant producers to manage brown rot disease by inducing plant systemic resistance and enhancing soil health indices (Seadh and El-Metwally 2015; Farag et al. 2017; Messiha and Elhalag 2019; Messiha et al. 2021). The implementation of good agricultural practises is critical in the context of sustainable management. These practises have been shown to be beneficial in terms of soil health, crop yield and disease control (Kharel et al. 2022, Chen et al. 2023).

In this context, DABs and extracts are rich in phycoelicitor compounds and are recognized as plant-defensive bio-stimulants (Shukla et al. 2021). Furthermore, algal-derived bioactives, including those from cyanobacterial species such as *Spirulina platensis* and seaweeds (marine macroalgae), have shown the potential to trigger plant antioxidant defence responses (Saber et al. 2018; Vasantharaja et al. 2019; Benítez García et al. 2020; El-Anany et al. 2020). Seaweeds are established as valuable source of extracts that act as biostimulant and protective agent (Ramkissoon et al. 2017; Hamed and Messiha 2018; Ali et al. 2019; Kergosien et al. 2023). They are being considered as a viable alternative to chemical fertilisers within the organic farming system.

Seaweeds typically contain a diverse array of inorganic and organic bioactive compounds, including, polyphenols, polysaccharides, laminarin, carrageenan, fucoidan, as well as phytohormones such as gibberellins, cytokinins, betaine, and auxins (Hassan et al. 2021; Shukla et al. 2021). Additionally, they exhibit variable contents of proteins, glycoproteins, lipids, and essential minerals like potassium (K), phosphorus (P), calcium (Ca),, and various trace elements (Van Oosten et al. 2017; Yakhin et al. 2017).

The use of seaweed extracts, whether applied as foliar sprays or incorporated into the soil, has been extensively studied and demonstrated to enhance plant growth, increase crop yields, and enhance plant resistance against a broad spectrum of plant pathogens. For instance, studies on tomatoes have illustrated these effects (Khan et al. 2009; Shukla et al., 2021).

Foliar application of *A. spicifera* extract at a concentration of 0.5 mg/mL has been shown to enhance plant resistance against *Phytophthora palmivora* infection in rubber trees (Pettongkhao et al. 2019). Additionally, it has demonstrated significant inhibitory effects on the growth of *Xanthomonas campestris* and *Alternaria solani* in tomatoes and sweet peppers (Ali et al. 2021). *Turbinaria* sp. and *Cystoseira* sp. have also exhibited antimicrobial properties against root rot disease, in tomato plants as reported in studies (Selvaraju & Vijayakumar 2016; Esserti et al. 2017).

Enhancing the soil suppressiveness by improving soil fertility indicators, such as available soil macronutrients (NPK), organic matter (OM%), and organic nitrogen (ON%)has been proven to be an effective and sustainable strategy for controlling disease in potatoes (Messiha 2006; Messiha and Elhalag 2019). Furthermore, promoting soil microbial biodiversity and increasing the ratio of antagonistic bacteria, such as fluorescent *Pseudomonads*, have been shown to significantly suppress *R. solanacearum* infecting potatoes (Messiha et al. 2019). The application of composted organic compounds, including plant compost, chicken manure, and animal manure, has also led to the suppression of *R. solanacearum* population and the stimulation of soil microbial biodiversity (Messiha et al. 2021). However, there is limited information available regarding soil biodiversity, soil fertility, and antagonist ratio influenced by algal application, whether to plants or soil.

The coastal areas host diverse array of seaweed species (Hamed and Messiha 2018; Hassan et al. 2021)and research has demonstrated their potential as effective alternatives for managing plant diseases. Seaweed-derived chemical compounds can activate plants’ defense mechanisms, thereby enhancing their resistance to pathogens. Investigations have revealed that seaweeds and their extracts influence various plant defense pathways, including hormonal signalling, antioxidant enzymes, oxidative burst, and stress-responsive genes (Bahmani et al. 2023). Nevertheless, there is a scarcity of studies focusing on the use of local marine macroalgae species as a natural resource for crop disease management, enhancing soil microbial diversity, and improving crop yield (Stiger-Pouvreau and Zubia 2020; Duarte et al. 2018). This research aims to explore the utilization of seaweed extracts from locally available seaweed-originated resources as organic inputs for integrated crop production. The study also investigates the mechanisms of action involved in the proposed phytoelicitor and biostimulatory activities of these seaweed extracts.

This research is part of an integrated program aimed at the 'Rehabilitation of the Nile Valley and Delta to produce brown rot-free potatoes qualified for exportation. The current study investigates the influences of different algal components, either as foliar spray or soil DABs amendments, on the seaweed species *A. spicifera*, *T. ornata*, *C. racemosa*, *C. myrica*, and the cyanobacterium *S. platenorsis* at two naturally infested locations with *R. solanacearum* and one non-infested location.

The objectives of this study are:

To examine the effects of algae-derived phytostimulatory activities on potato growth, yield, and quality.

To achieve sustainable disease suppression by improving soil fertility indices.

To enhance soil microbial biodiversity as an indicator of soil health across different locations.

## Materials and methods

### Algal species tested and biomass collected

*Spirulina platensis* cyanobacterium extract was obtained from the Algal Biotechnology Unit, National Research Centre (NRC), Dokki, Egypt. The marine macroalgal species of Rhodophyta, (*Acanthophora spicifera (Vahl) Borgesen*), Phaeophyta (*Turbinaria ornata (Turn.) J. Agardh* and *Cystoseira myrica (Gmelin), C. Agardh.* and Chlorophyta (*Caulerpa racemosa (Forssk.) J. Agardh*) were manually collected during low tides of the summer season 2021 at Marsa Allam (25° 4'11.41"N, 34°53’56.22") and Al-Quseir (26° 2'34.02"N, 34°18’51.51"E) seashore of the Red Sea, Egypt. To eliminate adherent pollutants and impurities, the algal specimens were washed with tap water and distilled water.

### Preparation of algal extract

The macroalgal specimens were air-dried in the shade for 72 hours. The algal biomass was ground and sieved using an electric mixer to produce a fine-grained powder texture. To make algal extract, biomass powder was periodically injected into a Soxhlet extractor equipped with a 500-ml capacity flask and a condenser. The extraction was carried out in a water bath for 12 hours using 300 ml of 95% methanol and 30 g of each powdered sample individually. The crude extracts were filtered through Whatman filter paper No. 1 and concentrated under reduced pressure in the rotatory evaporator (GG SENCO) until completely dry (Leelavathi and Prasad 2015). The dried crude extracts were kept at 4°C to investigate their antibacterial and phytochemical properties.

### Field experiments

The experiments were conducted at three different locations, two of which were naturally infested with *R. solanacearum*, location 1 at Kafr-Shukr village (30°34’02.0"N 31°1451.9E), location 2 at El-Mounira village (30°14’08.0"N 31°0736.7E), both on Kaliobeya governorate, and location 3 at Talia village, Minofyia governorate (30°1629.0N). These three locations are in the Nile Delta’s old districts, and the soil type is loamy clay. The effect of various algal applications, either as foliar spray or soil amendment by DABs, on brown rot disease control in susceptible potato Spunta cultivar, was examined. The soil was prepared by ploughing at each location and then split into 15 lines (10 m in length, 30 cm between plants, and 90 cm between lines) to represent five treatments in three repetitions. A completely randomized block design was used. Around 30 tubers (local seed potatoes) were planted per line and dispersed equally throughout the three designated locations. As a nitrogen supply, urea (46.5% N) was utilized, corresponding to 90 kg N/acre (200 kg urea). The nitrogen dosage was split into three equal parts based on the phases of vegetative development, tuberization, and tuber bulking. Superphosphate (37% P_2_O_5_) was also supplied in three equal doses at a rate of 28 kg P/acre (80 kg P_2_O_5_). Potassium fertilizer (50% K_2_O) was applied in two equal doses of 100 kg K/acre (200 kg K_2_O) during the second and third potato development periods exclusively (to avoid tuberization delay).

The proposed treatments consisted of a control with no algal application and two foliar spray treatments with *Acanthophora spicifera* or *Spirulina platensis* at a concentration of 1 mg/L. Prior to planting, the tubers were soaked for 30 minutes and allowed to dry. The leaf foliar spray was applied twice at 30 and 45 days after planting.

Two DABs supplements were used: *Turbinaria ornata* and a combination of *Caulerpa racemosa* and *Cystoseira myrica* (in a 1:1). Each treatment involved applying 5 grams of the respective DAB at a depth of 10–15 cm along with seed tubers before irrigation. During the potato harvest stage, 5 fresh leaves were randomly selected from at least 5 plants in each treatment for plant analyses. For soil analysis, at least four separate soil samples, each weighing 0.5 kg, to represent distinct treatments. These soil samples were obtained using an auger at a depth of 15–25 cm below the soil surface. After collection, the samples were thoroughly mixed to ensure homogeneity. Soil analyses including measurements oforganic matter (OM%) and available NPK were conducted on samples from each treatment.

### Potential of algal treatments on disease suppression

In the study, three soil samples, each weighing 10 grams, from the samples intended for chemical analysis. These samples were extracted using 0.05 M phosphate buffer (PB) solution. Subsequently, the plating was performed using two sets of plates with SMSA medium. The aim of this plating was to determine the number of colony-forming units (CFU) per gram of soil and tissues. To confirm the identity of the colonies, we conducted conventional PCR using specific primers RS-1-F and RS-1-R, which amplified a fragment of 718 base pairs (bp) (PM 7/21 (3) (2022)). Additionally, serological testing was performed using the Immunofluorescent Antibody Staining (IFAS) method as described by Janse in 1988. For the serological testing, a polyclonal antiserum for *R. solanacearum* (obtained from goat) was used. The antiserum was obtained from Loewe Biochemica GmbH (cat. No. 07356/01). Furthermore, Rabbit anti-Goat antibody conjugated with FITC (ab6737) from Abcam was employed as the secondary antibody for visualization.

For potato tuber analysis, we sampled all tubers from the treatment plots. Vascular tissue pieces were suspended in 40 ml of 0.05 M phosphate buffer (PB) and shaken for one hour. Afterward, the suspension was centrifuged at 4°C, and the resulting pellets were resuspended in 1 ml of 0.01 M PB. Aliquots of 100 µl were plated onto SMSA medium (Janse in 1988).

DNA extraction from the same potato extracts was performed using the Gene JET plant genomics kit, and DNA purification was carried out using the Thermo Scientific K0791 kit. To detect specific DNA sequences, we conducted a real-time fluorogenic PCR assay, specifically the TaqMan assay, following the protocol (Weller et al. 2000). Real-time PCR was conducted at the Central Laboratories, Faculty of Agriculture, Cairo University, using the Biorad cfx96 machine with RS-I-F, RS-II-R, and RS-P (FAM) primers (Weller et al., 2000).

### Effect of algae application on soil nutrients and organic matters

For soil analysis, available nitrogen (mineral N) was extracted using KCl and determined using the Technical Auto Analyzer II following Markus et al. (1982). Available phosphorus (P) was extracted using 0.5 N NaHCO_3_ as per Olsen et al. (1954) and measured calorimetrically according to Jackson (1962), with a KH_2_PO_4_ standard calibration curve. Available potassium (K) was extracted using ammonium acetate solutions per Jackson (1962) and measured using a Jenway flame photometer (PfP7, UK) with a KCl standard curve.

Organic matter (OM) content was determined using the Walkely and Black (1934) method. In this method, potassium dichromate was reduced by organic carbon (OC) compounds in soil samples, and unreduced dichromate was used to determine OC content through oxidation-reduction titration with ferrous ammonium sulfate

. The analysis was carried out at the Soil, Water, and Environmental Research Institute (SWERI) of the Agricultural Research Centre in Giza, Egypt.

### Assessment of macronutrient uptake (NPK) and potato yield

The dried leaves (0.5 gm) were digested in 10 mL concentrated H_2_SO_4_ at 400 ^o^ C in Kjeldahl flasks. Then 1 ml of mixture H_2_SO_4_: HClO_4_ (2:1) was added to plant digestate for bleaching. Total nitrogen (N) was determined using Kjeldahl method. Whereas, phosphorus (P) and potassium (K) contents were determined by spectrophotometer and flame photometer methods as described by Jackson (1973). The analysis was carried out at the Soil, Water, and Environmental Research Institute (SWERI) of the Agricultural Research Centre in Giza, Egypt.

The average of tuber weight per plant (average of three plants per line) along with the total weight of each line was determined (three lines per treatment)

### Determination of antioxidants

Total flavonoids (querectin equivalent) were estimated using Dowd’s method as modified by Arvouet-Grand et al. (1994). One ml of Aluminium chloride (2%) in methanol was mixed with an equal volume of the plant extract (2 mg). The absorbance reading was adjusted at 415 nm, after 10 min against a blank sample containing one ml of plant extract with one ml of methanol without Aluminium chloride. The flavonoids concentration was calculated using the following equation deduced from the standard quercetin curve:

Absorbance = 0.0258 quercetin (µg) – 0.0060 (R^2^: 0:9987).

The phenolic compounds (gallic acid equivalent) were determined by the Folin-Ciocalteu (FC) spectroscopic method as described by Singleton et al. (1999). One hundred microliters of leaf extract samples were reacted with 750 µl diluted Folin-Ciocalteu reagent (1:10) for 5 min. The solution was mixed with 750 µL NaHCO_3_ for another 90 min at room temperature in the dark. The absorbance of the developed bright blue color of phosphomolybdenum and phosphotungsten complexes, which resulted from the reaction of phenolic compounds with FC oxidants, was determined photometrically at 725 nm (Uv/ Vis Analytikjena spectro D250 Germany). Readings were generated using the standard curve of gallic acid equivalents, GAE/g (dry weight).

The total antioxidant capacity (ascorbic acid equivalent) was determined by phosphomolybdenum spectroscopic method as described by Prieto et al. (1999). One ml of each extract (0.5 mg/ ml) was mixed with 3 ml reagent solution (4 mM ammonium molybdate, 28 mM sodium phosphate and 0.6 M H_2_SO_4_). The mixture was heated up to 95°C for 2.5 hours, and then cooled down to room temperature. The absorbance degree of green colour, due to phosphomolybdenum complex formation, was determined at 695 nm. Total antioxidant capacity was expressed as ascorbic acid equivalent. The analysis was carried out at the Regional Centre for Food & Feed (RCFF) of the Agricultural Research Centre in Giza, Egypt.

### Correlation analysis between different parameters

Heat map and hierarchical clustering analysis of the different soil and plant characteristics were achieved by Pearson distance metric of the Multi Experiment Viewer (MeV)™ 4 software package (version 4.5, Boston, MA, USA)

Correlation analyses were performed on the combined data derived from the three different locations to study the relations between different tested parameters. Additionally, Principal Component Analysis (PCA) was carried out to assess the distribution of parameters across different locations and treatments.

### Bacterial community analyses in the rhizosphere

Additional analysis was conducted on the treatment involving the mixed DABs due to their superior suppressive effect. For soil DNA extraction, the DNeasy® PowerSoil® Kit (Qiagen GmbH, Germany, Cat. No. 12888-100) was used. DNA yield and purity were assessed using a NanoDrop (ND-1000 spectrophotometer), and only DNA samples with a purity ratio between 1.8 and 2 (measured at A260/A280 nm) were selected for metagenome analysis. Bacterial 16S amplicon sequencing was performed by Macrogen in South Korea using Illumina technology, following the procedure outlined in Messiha (2023).

### Statistical analysis and bioinformatics

Statistical analyses were performed using SPSS v. 23. Tukey multiple comparisons with Tukey’s honestly significant difference (HSD) test at a 95% confidence level and Dunnett’s t-test (two-sided) were conducted to compare multiple groups against the control group. Multivariate tests, specifically Wilks’ Lambda, were utilized to assess the effects of different treatments on the tested parameters. The data from multiple locations were pooled, and separate analyses were performed for each location using SPSS v. 23.

Heat map and hierarchical clustering analysis for different soil characteristics and bacterial species were achieved by Pearson distance metric of the Multi Experiment Viewer (MeV)™ 4 software package (version 4.5, Boston, MA, USA)

Principal Component Analysis (PCA) and data visualization were performed using R software, version 4.3.0., with the assistance of the following packages: "vegan," "permute," "lattice," "ggplot2," "tidyverse," "factoextra," and "ggrepel."

For testing the bacterial soil biodiversity, the raw reads were processed using QIIME2, version 1.9.1, to generate a table of operational taxonomic units (OTUs). Statistical analysis and visualization were conducted on a locally installed SHAMAN server. Bar plots for each diversity index were created in R v. 4.3.0 using the “ggplot2” and “patchwork” packages.

## Results

### Effect of algae application on potato brown rot suppression

**Fig. 1:**
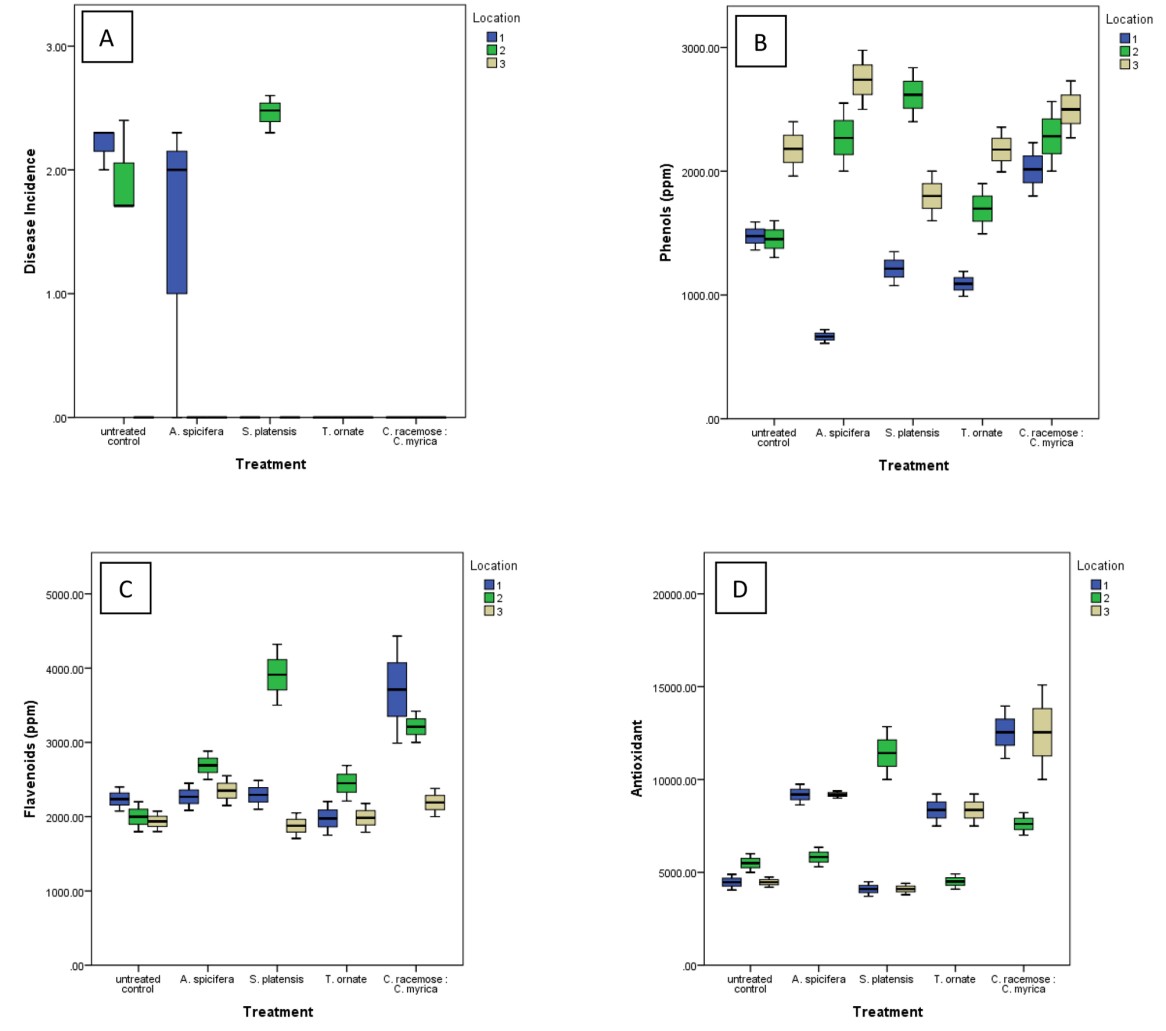
The effect of the different algal treatments on **(A)** disease incidence, **(B)** phenols, **(C)** flavonoids and **(D)** antioxidants of potato Spunta, at the harvest stage, cultivat in different locations naturally infested with *Ralstonia solanacearum*. Data are expressed as the mean of 3 independent replicates at the 3 different locations

**Fig. 2:**
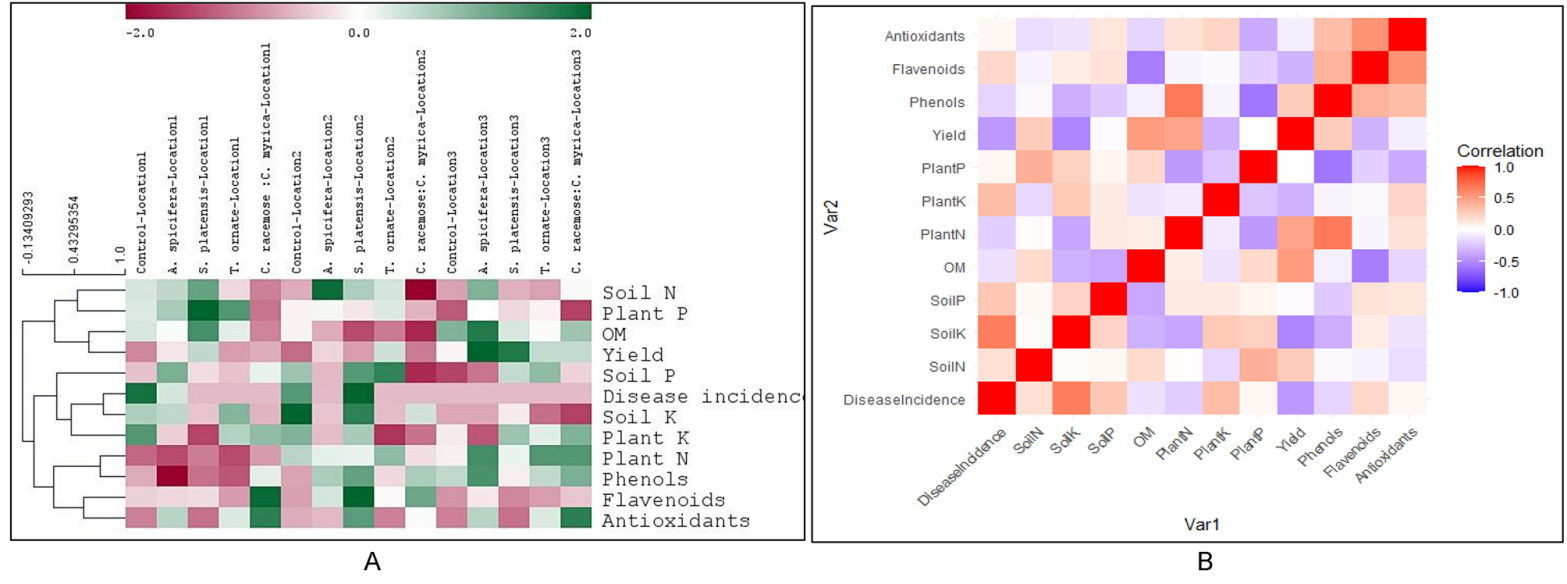
**(A)** Heat map and hierarchical clustering based on the soil, plant analysis, yield and disease incidence of three independent locations (1, 2 and 3) treated with different algal amendments (Untreated control, algal extracts (*Acanthophora spicifera*, *Spirulina platensis)*, dried algal biomasses (DABs) of *Turbinaria ornate* and a mixture of *Caulerpa racemosa* and *Cystoseira myrica* (1:1)) at the harvest stage (120 days). The patterns shown in the heat map indicate the average of three replicates for each parameter. Lower and higher concentrations were denoted by red colors and green respectively. The analyses were achieved by Pearson distance metric of the Multi Experiment Viewer (MeV)? 4 software package (version 4.5, Boston, MA, USA). **(B)** Correlation analysis of multiple factors for pooled data from the three different locations. SoilN, SoilP and SoilK = Nitrogen (ppm), Phosphorous (ppm) and Potassium (ppm) respectively. PlantN, PlantP and PlantK = N%, P% and K% respectively Phenols, Flavenoids and Antioxidants (ppm) Antioxidants = Total Antioxidant Capacity The analysis was carried out in R (4.3.0) with the “ggplot2” and “reshape2” packages

Disease incidence for potato soil and tubers treated with different algal extracts, or DABs, compared to their untreated controls is reported as follows:

Location 1: The population of the pathogen in the soil was determined to be 1.82 ± 1.22 (log10 CFU + 1)/g (mean ± standard error). The pathogen was also detected in potato extracts from tubers grown in the untreated plots, with a population of 2.22 ± 1.52 CFU/ml and a cycle threshold (CT) value of 23.4. For tubers treated with *A. spicifera*, the pathogen population in the soil was 2.00 ± 1.46 CFU/g, and 2.00 ± 1.76 CFU/ml (CT = 26.72) were recovered from the potato tubers. Tubers treated with *S. platensis* had a pathogen population in the soil of 1.92 ± 1.64 CFU/g, and the pathogen was below the detection level in the potato tubers. The pathogen was undetectable in both soil and tubers treated with DABs (Table 1, Fig. 1A, and Fig. 2A).

**Table 1:** Potato brown rot incidence under different algal treatments conditions

	Location 1					Location 2						
Treatment	Soil		Tubers			Soil		Tubers				
	(CFU/g)^3^ **SMSA**	IFAS^4^	SMSA	IFAS	RT-PCR^5^	(CFU/g) **SMSA**	IFAS	SMSA	IFAS	RT-PCR		
Untreated soil (control)	1.82 ± 1.22	+ve	2.22 ± 1.52	+ve	23.4	3.07 ± 2.83	+ve	2.07 ± 1.82	+ve	34.4		
*A. spicifera* ^1^	2.00 ± 1.46	+ve	2.00 ± 1.76	+ve	26.72	2.12 ± 1.95	+ve	0.00 ± 0.00		Und.		
*S. platensis* ^1^	1.92 ± 1.64	+ve	0.00 ± 0.00		Und**.	2.37 ± 2.12	+ve	2.48 ± 1.76	+ve	22.03		
*T. ornate* ^2^	0.00 ± 0.00		0.00 ± 0.00		44.05*	0.00 ± 0.00		0.00 ± 0.00		Und.		
*C. racemose : C. myrica*	0.00 ± 0.00		0.00 ± 0.00		Und.	0.00 ± 0.00		0.00 ± 0.00		Und.		
*(mix 1:1)* ^*2*^												

^1^ Algal extract ^2^ dried algal biomass (DAB) ^3^Log10 (Mean ± SE)

^4^IFAS ((Antiserum (anti-goat) and Rabbit anti-Goat antibody conjugated with FITC): a confirmatory test for typical colonies developed on SMSA

^5^Real-time PCR on *R. solanacearum* (RS-I-F, RS-II-R) and the fluorescent signal generated by RS-P (FAM) (Weller et al. 2000) using the Biorad cfx96 machine, real-time PCR.

PC is *R. solanacearum* race 3 biovar 2 Phylotype II, sequevar 1 culture, previously isolated and identified (Hamed and Messiha 2018)

*CT > 40 is considered negative, ** undetermined CT values

Location 3: The pathogen was undetectable in all treatments.

SMSA: South Africa Selective Media (Anonymous 1998). IFAS: Immunofluorescence Antibody Staining. (Janse 1988).

Location 2: The population of the pathogen in the soil was determined to be 3.07 ± 2.83 CFU/g. The pathogen was also detected in potato extracts from tubers grown in the untreated plots, with a population of 2.07 ± 1.82 CFU/ml (CT = 34.4). For tubers treated with *A. spicifera*, the pathogen population in the soil was 2.12 ± 1.95 CFU/g, and it was below the detection level in the potato tubers. Tubers treated with *S. platensis* had a pathogen population in the soil of 2.37 ± 2.12 CFU/g and 2.48 ± 1.76 CFU/ml (CT = 22.03) in potato tubers. The pathogen was undetectable in both soil and tubers treated with DABs. The IFAS test was used to confirm typical colonies recovered on SMSA (Table 1).

Multivariate analysis revealed significant differences in the population of the pathogen in soil between different treatments (F = 4.9, Wilks’ Lambda *P* < 0.001). Dunnett t (2-sided) analysis revealed a significant difference between the untreated control and the two DABs treatments *P* = 0.005).

### Effect of algae application on soil properties

**Fig. 3:**
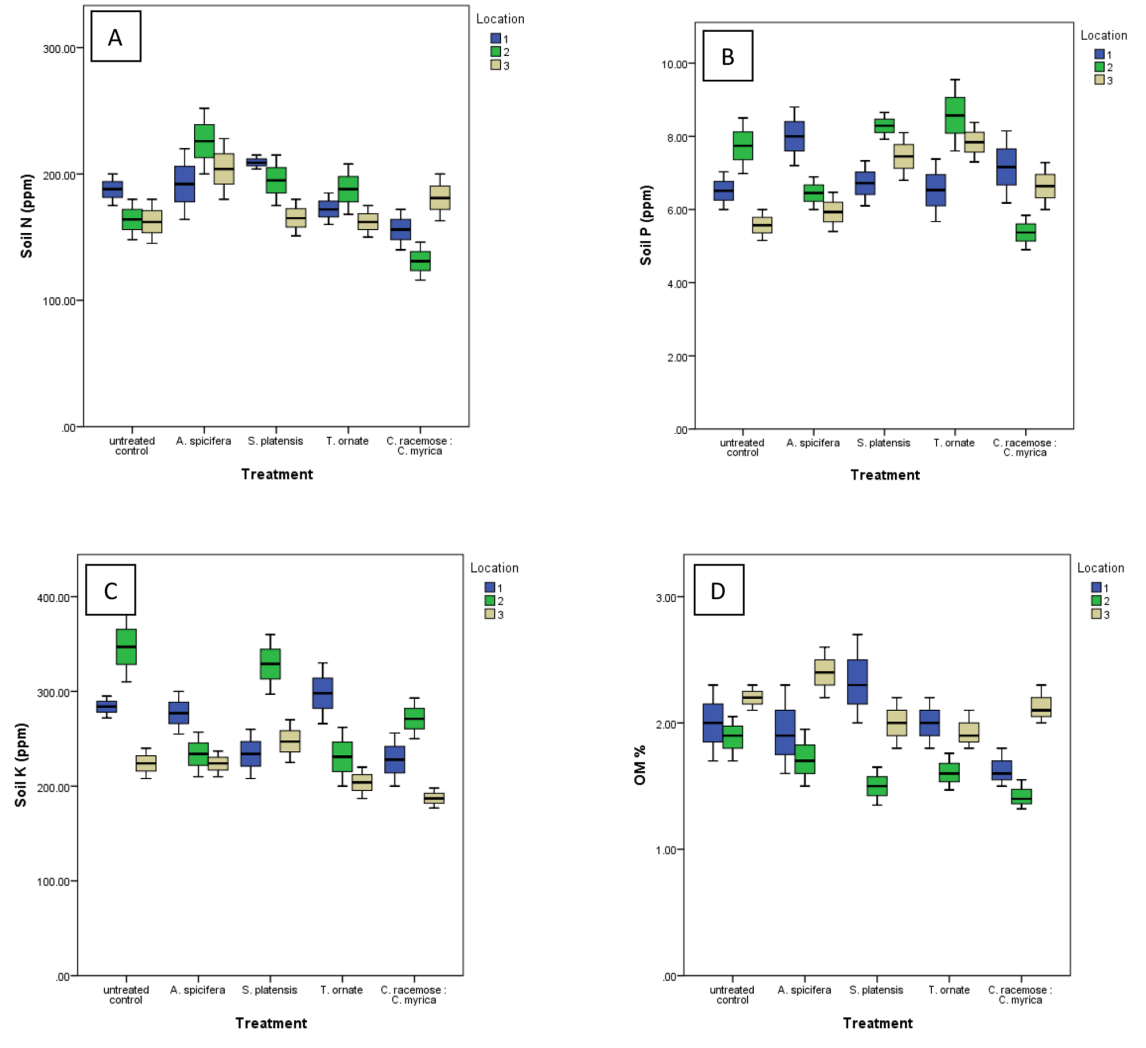
The effect of the different algal treatments on soil chemical properties; **(A)** available nitrogen (N ppm), **(B)** available phosphorus (P ppm), **(C)** available potassium (K ppm), **(D)** organic matter (OM%) at the harvest stage (120 days). Data are expressed as the mean of 3 independent replicates at the 3 different locations

The soil fertility index, represented by macronutrient elements (available NPK) content, and organic matter (OM%) were studied for different amendments as compared to the untreated control (Fig. 3). Multivariate analysis revealed significant differences in soil N between different treatments (F = 6.6, Wilks’ Lambda *P* < 0.001). The foliar application of *A. spicifera* (1.0 mg/L) showed the highest increases in soil N content in general (*P* = 0.006) (Figs. 2A and 3A).

On the contrary, different treatments didn’t affect soil P. Exceptionally, *C. racemosa* & *C. myrica* mixture (DABs mix) significantly decreased soil P (*P* = 0.004) at location 2. Meanwhile, *S. platensis* and *T. ornate* induced soil P at location 3 (*P* = 0.015 and *P* = 0.004 respectively) (Figs. 2A and 3B).

*C. racemosa* & *C. myrica* mixture (DABs mix) caused a significant decrease in soil K (*P* = 0.047) in all locations (*P* = 0.065, *P* = 0.033, and *P* = 0.062) for Locations 1,2, and 3, respectively. *A. spicifera* and *T. ornate* caused a similar decrease in Location 2 (*P* = 0.03 and *P* = 0.002) (Figs. 2A and 3C).

*C. racemosa* & *C.* myrica DAB mixture caused a decrease in OM being significant at location 2 (*P* = 0.022) (Figs. 2A and 3D).

### Effect of algal application on macronutrient uptake and potato yield

**Fig. 4:**
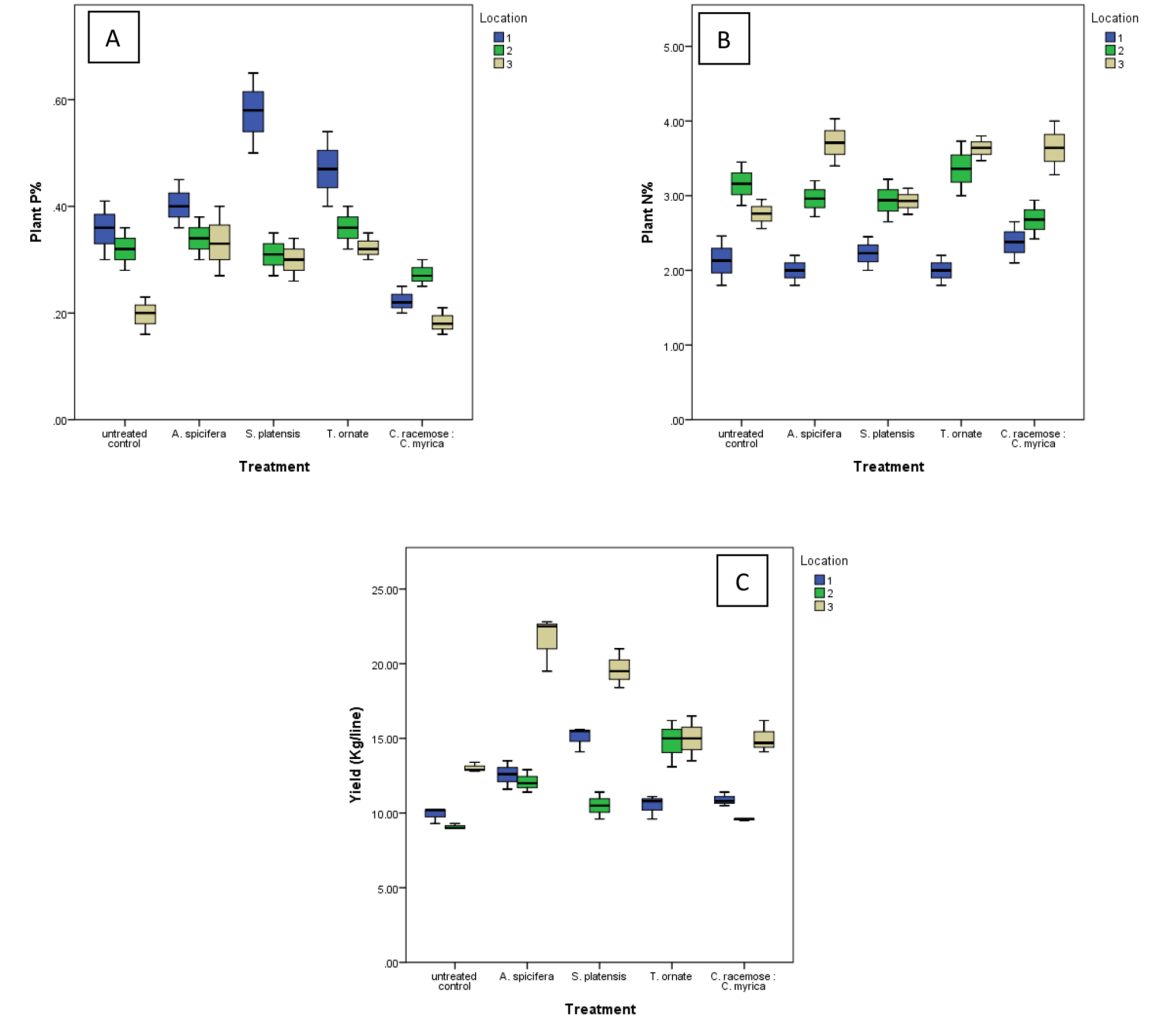
The effect of the different treatments on the nutritive status of the potato Spunta; leaves **(A)** N-uptake (N %), **(B)** P-uptake (P %), and **(C)** potato yield at the harvest stage (120 days). The potato plant was cultivated in the three different locations naturally infested with *Ralstonia solanacearum*. Data are expressed as the mean of 3 independent replicates at the 3 different locations

As essential elements for plant growth and biomarkers of plant health, nitrogen, phosphorus, and potassium (NPK) uptake were determined in leaves after 75 days of planting. Results in Figs. 2A and 4 revealed that algal treatment had a promotive effect on N and P uptake, particularly at location 3. For instance, *A. spicifera* increased N and P uptake (34%, *P* = 0.003 and 65%, *P* = 0.007) respectively as compared to the untreated control. The foliar spray of *S. platensis* upgraded P uptake at location 1 by 61%, *P* = 0.003 and by 50%, *P* = 0.035 at location 3. A similar finding was observed for *T. ornata* which caused a similar increase (32%, *P* = 0.006 and 60%, *P* = 0.011) for N and P respectively, at Location 3. Meanwhile, the DAB remarkably increased N-uptake by 32%, *P* = 0.006 at the same location.

In general, a foliar spray of *A. spicifera* and the cyanobacterium extract of *S. platensis* showed a significant increase in tuber yield (*P* = 0.015 and *P* = 0.027). In particular, *A. spicifera* caused a significant increase in tuber production (26% (*P* = 0.004), 33% (*P* = 0.006) and 57% (*P* < 0.001) for locations 1, 2, and 3 respectively. *S. platensis* caused a significant increase in tuber production (51% and 52%, *P* < 0.001) for locations 1 and 3, respectively. *T. ornate* caused a significant increase in tuber production (61% (*P* < 0.001)), for location 2 only.

Multivariate analysis revealed significant differences in plant P between different treatments (F = 5.9, Wilks’ Lambda *P* = 0.001). Dunnett t (2-sided) analysis revealed a significant increase in *S. platensis* treatments as compared to the untreated control *P* = 0.053. Meanwhile, only a trend of significant decrease was recorded for plant K (F = 2.4, Wilks’ Lambda *P* = 0.06) with a significant decrease for *A. spicifera* treatment as compared to the untreated control *P* = 0.015 (Supp. Figure 1).

A significant differences in potato yield between different treatments (F = 3.4, Wilks’ Lambda *P* = 0.017). Dunnett t (2-sided) analysis revealed a significant increase in *A. spicifera* and *S. platensis* treatments as compared to the untreated control *P* = 0.015 and *P* = 0.027 respectively.

### Effect of algal treatments on plant antioxidants

Field studies and multivariate analysis revealed a significant increase in flavonoid contents and total antioxidant capacity (TAC) among different treatments (F = 4.3 and 7.6 Wilks’ lambda *P* = 0.006 and *P* < 0.001, respectively). Dunnett t (2-sided) analysis revealed a significant increase in flavonoids for the mixed DABs treatments as compared to the untreated control *P* = 0.004. Also, a significant increase in TAC for the *A. spicifera* and the mixed DABs treatments as compared to the untreated ontrol (*P* = 0.025, and *P* < 0.001), respectively.

The foliar spray with *A. spicifera* also increased phenolic compounds and flavonoid contents at location 2 as compared to the untreated control (56%, *P* = 0.005 and 35%, *P* = 0.029, respectively), and only the phenolic compounds at location 3 (26%, *P* = 0.031). That may respectively lead to an upgrad in total antioxidant capacity by 106%, *P* = 0.003 at locations 3 (Figs. 1 and 2A).

*S. platensis* foliar application showed also a similar stimulating effect on phenols, flavonoids and total antioxidant potential at location 2 by 80%, *P* < 0.001; 96%, *P* < 0.001; and 103%, *P* < 0.001, respectively.

The plant antioxidants of *T. ornata* were differentially induced. TAC was increased by 87% (*P* = 0.019) only at location 3.

In conclusion, field studies revealed that mixed DABs markedly increased flavenoids and TAC in potato leaves, which was typically higher at locations 1 and 3 (three times as compared to the untreated control, *P* < 0.001) and 38%, *P* = 0.001 higher for location 2. This effect was in accordance with the evidenced accumulation of phenolic compounds and flavonoid contents at location 1 with 37%, *P* = 0.009 and 66%, *P* = 0.002, respectively as compared to the untreated control. Similar results were recorded for location 2, with 57%, *P* = 0.004 and 61%, *P* = 0.001 increases for phenolic compounds and flavonoid contents, respectively. No significant difference was recorded for location 3 (non-infested soil).

### Correlation analysis between different parameters

Figure 2B illustrates the correlation analysis between the various tested parameters in relation to different locations and algal treatments.

The results of the analysis demonstrated a negative relationship between disease incidence and potato yield (correlation coefficient: -0.44, *P* = 0.002). In contrast, there were positive relationships between disease incidence, soil K (+ 0.65, *P* < 0.001), plant K (+ 0.346, *P* = 0.02), and soil P (+ 0.29, *P* = 0.002).

Potato production from one side had a significant positive correlation with soil N (+ 0.26, *P* = 0.008), plant N (+ 0.47, *P* = 0.001), and OM (+ 0.51, *P* < 0.001) from the other side (Fig. 2B).

It is important to highlight that there was a significant negative correlation between OM on one side and soil P (-0.38, *P* = 0.01) and soil K (-0.33, *P* = 0.03) on the other.

It was interesting to notice that phenolic compounds were correlated positively with plant N (+ 0.67, *P* < 0.001) and negatively with plant P (-0.59, *P* < 0.001) and soil K (-0.35, *P* = 0.018). Flavonoids, on the other hand, were negatively correlated with OM (-0.56, *P* < 0.001) and potato yield (-0.32, *P* = 0.03). TAC was also shown to be negatively correlated with plant P (-0.36, *P* = 0.014).

A significant positive correlation was recorded between phenolic compounds, flavonoids, and TAC.

In Supp. Figure 2, the results of the PCA analysis are presented. The plot illustrates a clear distinction between location 3 (non-infested) and the other two locations (infested), indicating distinct patterns. The untreated controls for the infested locations exhibited proximity to each other but were distinct from the un-infested location. Furthermore, the PCA plot revealed that the mixed DABs (*Caulerpa racemose: Cystoseira myrica*) treatment for different locations clustered together, indicating similar response patterns.

### Microbial community in the soil rhizosphere following algal application

**Fig. 5:**
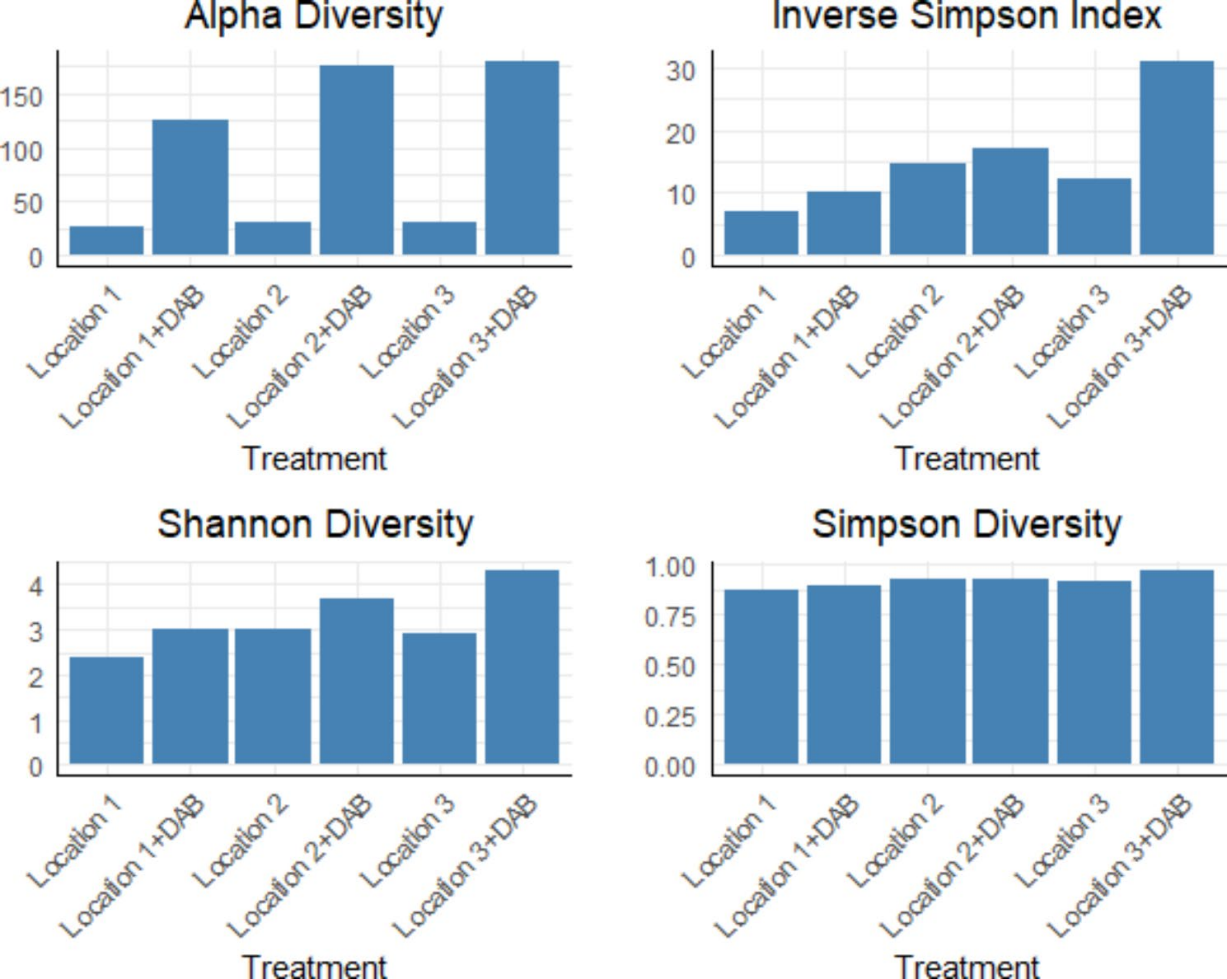
Effect of dried algal biomass amendment using *Caulerpa racemose: Cystoseira myrica*, mixture 1:1 on rhizosphere bacterial biodiversity at the harvest stage of potato plant cultivated in three independent locations naturally infested with *Ralstonia solanacearum*. DAB: mixed dried algal biomass (*Caulerpa racemose: Cystoseira myrica*). Five grams of each DAB was applied at 10–15 cm deep with seed tubers. The bacterial 16S amplicons were sequenced using Illumina technology. The sequencing platforms employed were MiSeq

The mixed DABs treatment exhibited the highest suppressive effect compared to other applications, and it was distinctly separated from all other treatments. This distinct separation and superior performance led to the selection of the mixed DABs treatment for further microbial community analysis. Furthermore, the mixed DABs treatment significantly enhanced soil microbial biodiversity, as evidenced by an increase in alpha diversity measures, including the Inverse Simpson index, Shannon diversity index, and Simpson diversity index, when compared to untreated soil in all three locations (Fig. 5).

**Fig. 6:**
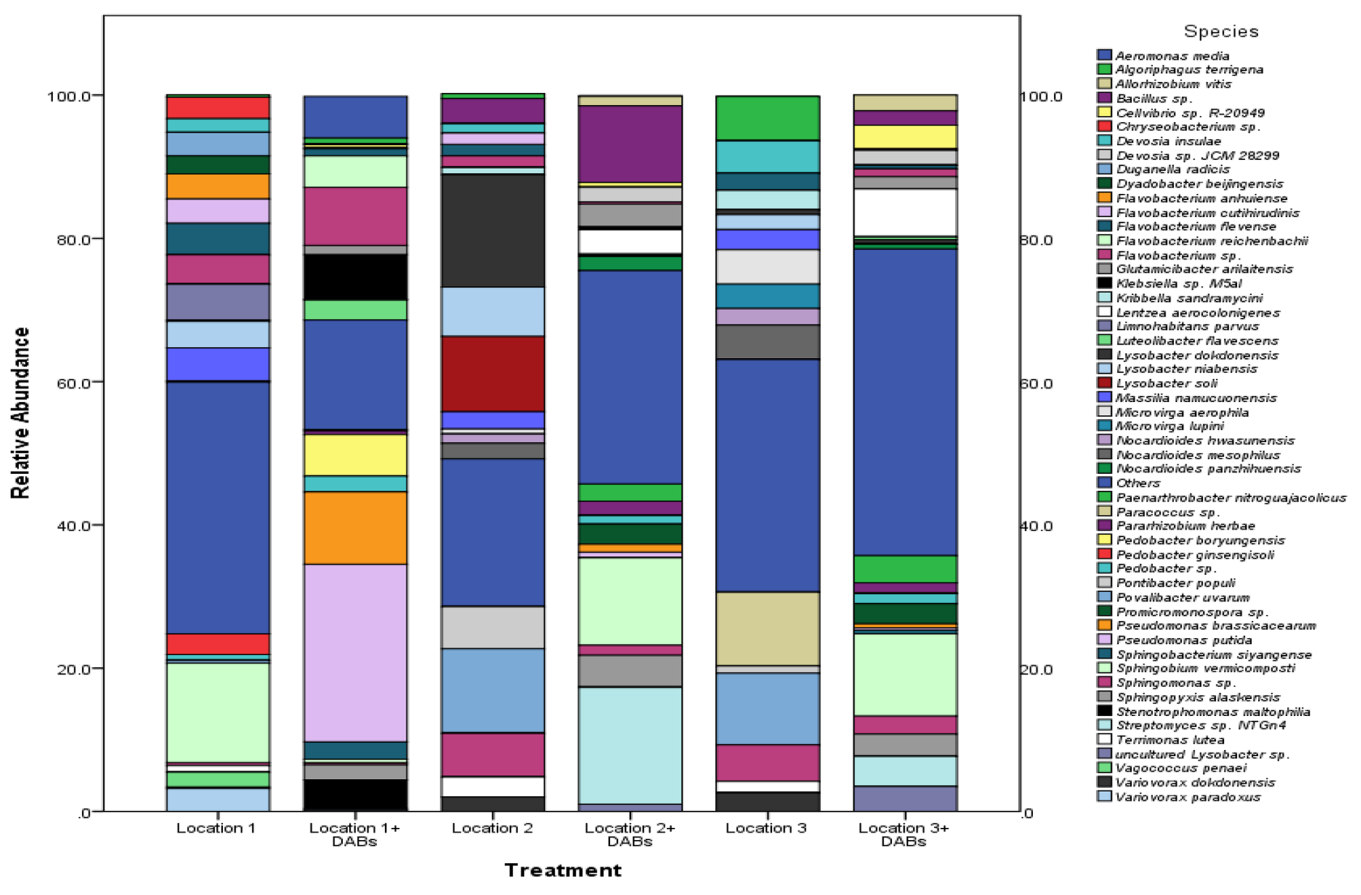
The most abundant bacterial species in potato soil rhizosphere, at the harvest stage, of potato plant treated with dried algal biomass of *C. racemosa* and *C. myrica* (1:1) compared to untreated control conditions. The potato plant was grown in three independent locations naturally infested with *Ralstonia solanacearum*. Based on ILLUMINA Miseq data

**Fig. 7:**
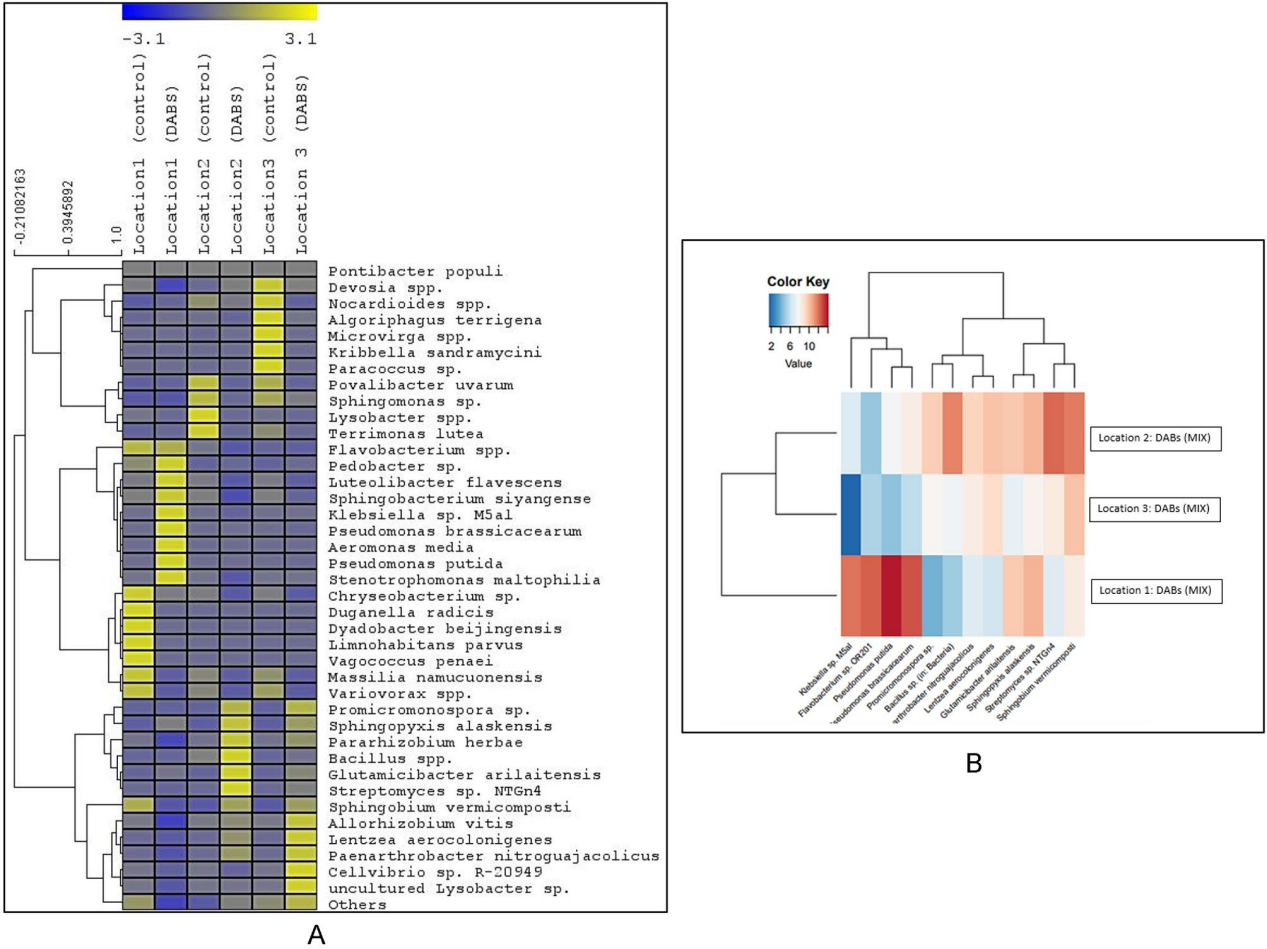
**(A)** Heat map and hierarchical clustering based on the most abundant bacterial species in different potato rhizosphere (untreated control and treated with the mix dried algal biomasses (DABs) of *Caulerpa racemosa* and *Cystoseira myrica* (1:1) at locations 1, 2 and 3 based on ILLUMINA Miseq data. **(B)** Heat map depicting the distribution of the most abundant bacterial species in different potato rhizosphere treated with the mix dried algal biomasses (DABs) of *Caulerpa racemosa* and *Cystoseira myrica* (1:1) at locations 1, 2 and 3 based on ILLUMINA Miseq data. Bacterial species: *Klebsiella* sp. M5al, *Flavobacterium* sp. OR201, *Pseudomonas putida*, *Pseudomonas brassicacearum*, *Promicromonospora* sp., *Bacillus* spp., *Paenarthrobacter nitroguajacolicus*, *Lentzea aerocolonigenes*, *Glutamicibacter arilaitensis*, *Sphingopyxis alaskensis*, *Streptomyces* sp. NTGn4 and *Sphingobium vermicomposti*

Figures 6 and 7 provide a summary of the most abundant bacterial species in soils from the three locations, both untreated and treated with mixed DABs. The mixed DABs treatment led to increased abundances of *Glutamicibacter arilaitensis* and *Sphingopyxis alaskensis* in all locations. Location 1 showed the highest abundance of *Flavobacterium* sp., which was further enhanced by the mixed DABs treatment. Additionally, the treatment resulted in increased abundances of *Klebsiella* sp., *Pseudomonas putida*, and *P. brassicacearum*, primarily in location 1, with a lesser effect in the other two locations. Notably, these four species exhibited correlations with each other (Fig. 7).

In locations 2 and 3, the mixed DABs treatment resulted in increased abundances of *Lentzea aerocolonigenes, Paenarthrobacter nitroguajacolicus, Bacillus* sp., *Streptomyces* sp., and *Promicromonospora* sp. In Location 1, the mixed DABs treatment resulted in a significant decrease in the abundance of *Sphingobium vermicomposti*. Conversely, the abundance of *S. vermicomposti* noticeably increased when the mixed DABs were applied in the other two locations. Figure 6 clearly demonstrates a correlation between *S. vermicomposti* and *Streptomyces* sp. This correlation suggests a relationship or association between the abundance of these two bacterial species in the soil.

Moreover, a significant decrease in the abundances of *Variovorax* spp., *Lysobacter* spp., *Massilia namucuonensis, Nocardioides mesophilus*, and *Povalibacter uvarum* was observed in all locations.

## Discussion

### The effect of different seaweed extracts and dried algal biomasses (DABs) on disease suppressiveness and potato production

The commercialization of seaweed biomass and its derivatives in crop management systems has gained attention in recent years (Hamed et al. 2018; Shukla et al. 2021). In our study, *A. spicifera* demonstrated the most significant enhancement in potato crop production, both in naturally-infested and non-infested locations. The foliar application of *A. spicifera* extract positively influenced NPuptake, particularly in location 3 (non-infested area), which correlated with increased potato output (see Fig. 4). These findings align with the results reported by Garai et al. (2021) and Wadas & Dziugieł (2019). Previous research has also indicated that *A. spicifera* exhibits a phytostimulatory effect on tomato and sweet pepper development and yield components (Ali et al. 2021). Additionally, the application of *S. vulgare* and *A. spicifera* has been shown to significantly activate the biosynthesis of auxin (IAA), gibberellin (Ga2Ox), and cytokinin (IPT) genes (Ali et al. 2022).

Furthermore, our current research revealed that *A. spicifera* significantly increased the nitrogen (N) content in the soil, which may explain its conducive effect on bacterial wilt. The positive correlation between soil N and the survival of *R. solanacearum* has been previously addressed by Messiha et al. (2009).

This research demonstrates that the foliar application of *S. platensis* (1 mg/L) resulted in enhanced growth and yield of bio-fortified potatoes, particularly in locations 1 and 3. This improvement in potato growth and yield was associated with improved phosphorus (P) absorption at these specific locations. Similar positive effects have been observed in previous studies on potatoes and radishes, where the foliar application of *S. platensis* has led to positive outcomes in terms of crop growth and yield (El-Anany et al. 2020 and Godlewska et al. 2019). *S. platensis* is a cyanobacterial strain that is rich in micro and macroelements, as well as bioactive substances such as amino acids, carbohydrates, peptides, and phytohormones like gibberellins, auxins, and cytokinins (Godlewska et al. 2019). *Spirulina* filtrate/homogenate has been shown to enhance mineral content in plants by increasing sink strength, thereby impacting substrate flow, including minerals, within the plant promoting plant development (Khan et al. 2009 and Calvo et al. 2014). The biostimulant effect of seaweeds can be attributed to their bioactive contents, including the presence of phytoelicitors like salicylic acid, jasmonic acid, and ethylene which stimulate growth and productivity (Jayaraj et al. 2008; Khan et al. 2009; Ramkissoon et al. 2017; Ali et al. 2019).

In previous studies, the disease-suppressive effect of *S. vulgare* and *A. spicifera* was attributed to the sustainable induction of antioxidant defense enzymes activities, up-regulation of phenolic compounds, and biosynthesis of phytohormone (Ali et al. 2021). λ-carrageenan extract (0.5 mg/mL) of *A. spicifera* upgraded the expression of salicylic acid-dependent defense genes in rubber tree leaves against *Phytophthora palmivora* (Pettongkhao et al. 2019). A study by Ali et al. (2021) indicated that the application of *S. vulgare* and *A. spicifera* as foliar sprays induced transcript levels of the marker genes (PR-1a, PinII, and ETR-1) that contribute to defense signalling pathways. Nevertheless, in our study, these two algal extracts were ineffective in suppressing bacterial wilt. *PR-1a* is an acidic *PR* gene (Addy et al. 2012), which might explain why it is ineffective under the alkaline conditions of the Egyptian soils.

Our findings also revealed that using *T. ornate* as a DAB resulted in a significant increase in potato yield only at location 2, which was accompanied by a noticeable increase in soil P and plant N. This observation is consistent with the findings of Uthirapandi et al. (2018) and Karthik et al. (2020). Although *T. ornate* liquid extract had a distinct hormonal composition with a higher amount of phytohormones (e.g., auxin, gibberellin, and cytokinin). The use of liquid fertilizer derived from *T. ornate* increased plant growth and improved the biochemical composition of starch, glucose, protein, and chlorophyll *a* and *b* contents in *Ocimum sanctum* (Uthirapandi et al. 2018), as well as significantly improving the growth performance of *Raphanus sativus, Phaseolus vulgaris*, and *Vigna radiata* (Karthik et al. 2020).

In the present study, we observed that DABs of *T. ornate*, and a mixed dry mass of *C. racemosa* with *C. myrica* showed greater disease suppression when compared to foliar application. This mightbe due to the cumulative and synergistic effects of the qualitative and quantitative active ingredients of antibiotics, lytic enzymes, fatty acids, polyphenols and bioactive compounds that are naturally found in the DABs when compared to their individual liquid extracts. Also, the disease-suppressiveness associated with the mix of DABs was accompanied by a significant increase in flavonoids and total antioxidants (TAC). This finding aligns with observations in several plant species where, the aqueous and alcoholic extract of *Turbinaria conoides*, *Ulva reticulata* and *Cystoseira myriophylloides* have demonstrated effective antifungal effect against various plant phytopathogens, including *F. oxysporum*, *Rhizoctonia solani*, *Macrophomina phaseolina*, *Sclerotium rolfsii* and *Alternaria solani* (Selvaraju & Vijayakumar 2016; Karthik et al. 2020). Additionally, these seaweeds-derived extracts have inhibited the growth of *V. dahliae* and *Agrobacterium tumefaciens* in tomato plants (Esserti et al. 2017).

In general, there is a noticeable decrease in available potassium (K) in both soil and plant tissues after the application of algal extracts or dried algal biomasses (DABs). This phenomenon can be attributed to various factors, including elevated levels of soil calcium (Ca), salinity, and the presence of clay minerals. This proposition is in accordance with the postulation made by Castro and Gómez (2013). Furthermore, marine seaweed is well-known for its content of chelating agents, particularly alginate-metal ion combinations, which contribute to its capacity to chelate and bind metal ions in the soil. The formationof alginate-metal ion complexes by seaweed further reinforces its role as a chelating agent in agricultural systems (Battacharyya et al. 2015).

### Effect of algal amendments on soil bacterial diversity

Our results indicated that *C. racemosa* and *C. myrica* DABs enhanced soil health by upgrading the soil microbial community (Fig. 5) and induced pathogen suppression in two infested locations (Fig. 1). In fact, pathogen survival in soil is positively associated with total soluble organic matter and negatively influenced by bacterial diversity, as estimated from the results of denaturing gradient gel electrophoresis (DGGE) of eubacterial 16S rDNA directly extracted from soil (Messiha, 2006). Organic management and NPK fertilization were demonstrated to restore soil organic matter, improve soil quality, increase soil microbial biodiversity, and hence suppress the disease (Messiha et al. 2007; Luo et al. 2015). In our study, the application of mixed DABs increased the soil bacterial biodiversity. Previous reports indicated that seaweed alginates induce complex formations that absorb soil moisture and swell in size, which subsequently increases the soil water-holding capacity and aeration. This in turn encourages soil microbial growth and significantly contributes in enhancement of rhizosphere microbiome (Spinelli et al. 2010). For instance, soil application of *A. nodosum* extracts increased the rhizosphere bacterial biodiversity of pepper plants (Renaut et al. 2019) and altered the diversity of the soil fungal community (Wang et al. 2017).

The mixture of the two DABs, *C. racemosa* and *C. myrica* improved soil microbial communities shifting the soil bacterial biodiversity in the suppressive direction as follows:

The suppressiveness effect of the combination of *C. racemosa* and *C. myrica* DABs may be attributed to the increased abundance of *S. alaskensis* at the three locations. *S. alaskensis* is an oligotrophic marine bacteria that is considered a competitive species as addressed by Cavicchioli et al. (2003). Also, the mixture increased the abundance of *G. arilaitensis* at the three locations. *G. arilaitensis* is considered a plant growth-promoting agent under cooling conditions (Borker et al. 2021).

Location 1 exhibited the highest abundance of *Flavobacterium* sp., which was further increased by the mixed DABs treatment. This increase was accompanied by an increase in the abundances of *Klebsiella* sp., *Pseudomonas putida*, and *P. brassicacearum* (Fig. 6). These species are known biocontrol agents against bacterial wilt and other plant pathogens (Bahmani et al. 2021 and Kim et al. 2022). These species were less abundant in locations 2 and 3, where *Streptomyces* sp., *Bacillus* sp., and *Sphingobium vermicomposti* were prevalent, perhaps inhibiting them. Both *Streptomyces* sp. and *Bacillus* sp. are soil health indicators, as both of them are known biocontrol agents and drought-resistant (Köberl et al. 2013).

This algal dried mixture increased the abundances of *Lentzea* spp. and *Promicromonospora* sp. at locations 2 and 3 only, but not at location 1, where *Klebsiella* sp., *Flavobacterium* sp., *P. putida* and *P. brassicacearum* abundances were higher, which may inhibit *Lentzea* spp. and *Promicromonospora* sp. at this location. Both *Lentzea* spp. and *Streptomyces* spp. were more abundant in the mixed DABs-treated soil compared to the untreated control. Boron tolerance is shared by both bacterial taxa (Moraga et al. 2014), which may imply an increase in soil boron concentration as a result of DABs amendment (Miller et al. 2016). Moreover, *Promicromonospora* sp. genera are known for improving plant growth through Gibberellin production (Kang et al. 2012). *P. nitroguajacolicus* abundance was increased in the mixed DABs treated soil compared to the untreated control, which can be considered a soil health indicator. *P. nitroguajacolicus* increased the number of productive tomato plants, as addressed by Riva et al. (2021).

On the other hand, a significant decrease in some beneficial bacteria such as *Variovorax paradoxus, Lysobacter* spp., *Massilia namucuonensis, Nocardioides mesophilus*, and *Povalibacter uvarum rhizosphaere* was recorded in DABs-treated soil compared to the untreated in all locations.

*V. paradoxus* is known as a plant growth promoter (Han et al. 2011). *Lysobacter* spp are known as a potential source of novel antibiotics (Panthee et al. 2016). *Massilia* is known to produce violacein which has antibacterial, antiparasitic, and antiviral activities (Agematu et al. 2011)

## Conclusion

The foliar application of *A. spicifera* and *S. platensis* shows significant promise as a long-term potato-phycoelicitor for enhancing potato crop production. This enhancement was positively correlated with increased NP-uptake.

In contrast,, DABs demonstrate superior disease control capabilities, accompanied by substantial increases in flavonoids and total antioxidant capacity (TAC). Since the pathogen is soil-borne and DABs were incorporated into the soil, this may explain the disease suppressiveness in the soil. Furthermore, this suppressiveness is linked to increased soil bacterial biodiversity, notably an increased abundance of oligotrophic beneficial marine bacterial species.. Further research is needed to explore the synergistic effects of combining algal extracts with different types of dried algal biomass to maximize productivity while effectively suppressing diseases.

### Electronic Supplementary Material

Below is the link to the electronic supplementary material

Supplementary Material 1

Supplementary Material 2

## Data Availability

The data and material used during the current study are available from the author on reasonable request.

## Abbreviations

DAB: Dried algal biomass
TAC: Total antioxidant capacity
OM: Organic nitrogen
IFAS: Immunofluorescent Antibody Staining
SMSA: Selective Media South Africa
CCA: Canonical-correlation analysis analyses
PCA: Principal Component Analysis
