# Supplementary material for: Potential of algal-based products for the management of potato brown rot disease
Source: Bot Stud. 2023 Oct 16;64:29. doi: 10.1186/s40529-023-00402-y (PMC10579212; doi:10.1186/s40529-023-00402-y)
Supplement: Supplementary file 1 — Supplementary Material 1 [file 40529_2023_402_MOESM1_ESM.docx]

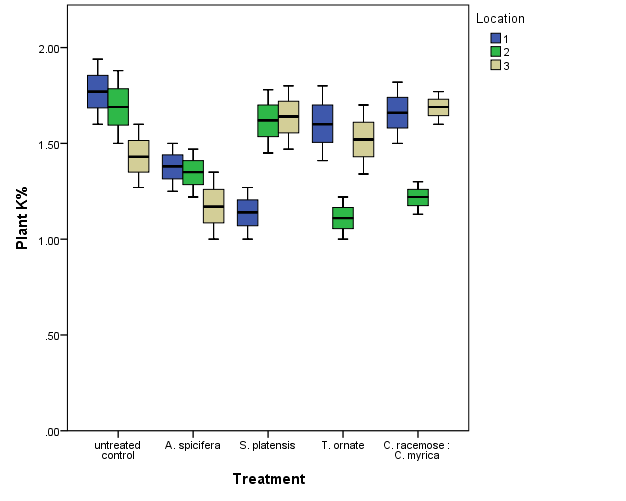


Supp. Fig 1. The effect of the different treatments on K-uptake (K %) for the different locations at the end of the experiment.

Data are expressed as the mean of 3 independent replicates at the 3 different locations
