# Supplementary material for: Potential of algal-based products for the management of potato brown rot disease
Source: Bot Stud. 2023 Oct 16;64:29. doi: 10.1186/s40529-023-00402-y (PMC10579212; doi:10.1186/s40529-023-00402-y)
Supplement: Supplementary file 2 — Supplementary Material 2 [file 40529_2023_402_MOESM2_ESM.docx]

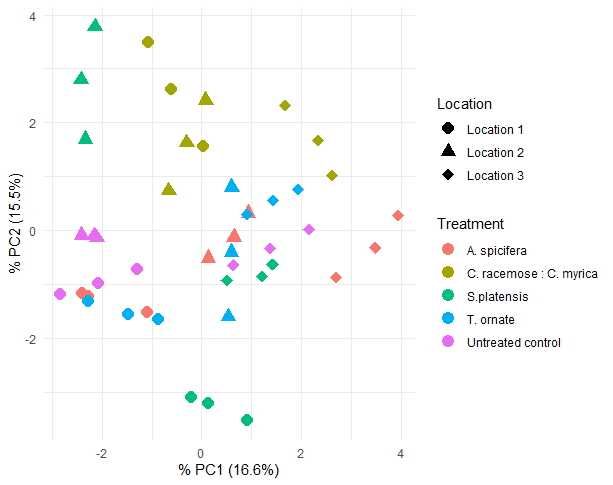


Supp. Fig. 2. Principal Component Analysis showing the influence of different algal treatments on potato growth parameters grown at 3 independent locations naturally infested with Ralstonia solanacearum.

The analysis was carried out in R (4.3.0) with the “tidyverse”, “factoextra” and “ggplot2” packages.
